# High throughput transcriptome analysis of lipid metabolism in Syrian hamster liver in absence of an annotated genome

**DOI:** 10.1186/1471-2164-14-237

**Published:** 2013-04-10

**Authors:** Roland Schmucki, Marco Berrera, Erich Küng, Serene Lee, Wolfgang E Thasler, Sabine Grüner, Martin Ebeling, Ulrich Certa

**Affiliations:** 1 F. Hoffmann-La Roche AG, pRED, Postfach, Basel, 4070, Switzerland; 2 Tissue Bank under the authority of HTCR, Visceral, Transplantation, Vascular and Thoracic Surgery, Grosshadern Hospital, Ludwig Maximilians University, Munich, 81377, Germany

**Keywords:** Next generation sequencing, Liver transcript profiling, Syrian hamster, Dyslipidaemia

## Abstract

**Background:**

Whole transcriptome analyses are an essential tool for understanding disease mechanisms. Approaches based on next-generation sequencing provide fast and affordable data but rely on the availability of annotated genomes. However, there are many areas in biomedical research that require non-standard animal models for which genome information is not available. This includes the Syrian hamster *Mesocricetus auratus* as an important model for dyslipidaemia because it mirrors many aspects of human disease and pharmacological responses. We show that complementary use of two independent next generation sequencing technologies combined with mapping to multiple genome databases allows unambiguous transcript annotation and quantitative transcript imaging. We refer to this approach as “triple match sequencing” (TMS).

**Results:**

Contigs assembled from a normalized Roche 454 hamster liver library comprising 1.2 million long reads were used to identify 10’800 unique transcripts based on homology to RefSeq database entries from human, mouse, and rat. For mRNA quantification we mapped 82 million SAGE tags (SOLiD) from the same RNA source to the annotated hamster liver transcriptome contigs. We compared the liver transcriptome of hamster with equivalent data from human, rat, minipig, and cynomolgus monkeys to highlight differential gene expression with focus on lipid metabolism. We identify a cluster of five genes functionally related to HDL metabolism that is expressed in human, cynomolgus, minipig, and hamster but lacking in rat as a non-responder species for lipid lowering drugs.

**Conclusions:**

The TMS approach is suited for fast and inexpensive transcript profiling in cells or tissues of species where a fully annotated genome is not available. The continuously growing number of well annotated reference genomes will further empower reliable transcript identification and thereby raise the utility of the method for any species of interest.

## Background

Clinical dyslipidaemia is defined as an abnormal concentration of lipids in the blood circulation caused by cholesterol-rich diet or exposure to elevated insulin levels. It is long known that elevated High-density lipoprotein-cholesterol (HDL-c) levels are associated with reduction of the risk of atherosclerotic cardiovascular diseases [1]. This effect is mechanistically related to reverse cholesterol transport (RCT), a process that mediates excretion of excess HDL cholesterol from peripheral tissues to the liver ending ultimately in the faeces [2]. Cholesteryl ester transfer protein (CETP) plays a central role in this process and it was recognized that pharmacological inhibition of CETP mediated cholesterol transport would raise HDL-c levels resulting in protection from coronary heart disease. The Syrian hamster *Mesocricetus auratus* has become an important animal model for pre-clinical dyslipidaemia research because HDL-c levels are raised in response to drugs designed for human target proteins [3]. In contrast to mice and rats, CETP is expressed in hamster liver which explains raise of serum HDL levels in response to the CETP inhibitor Anacetrabip [4].

The Illumina human body map 2.0 project provides high-quality RNAseq based gene expression data for major human organs using pooled samples from healthy donors including liver (http://www.ncbi.nlm.nih.gov/geo/). This database allows a comparative analysis of liver gene expression between humans and model organisms such as the primate *Macaca fascicularis* where a microarray based expression database of 36 liver samples became recently available as a part of a genome sequencing project [5].

Currently, lack of a fully annotated genome for *M*. *auratus* prevents development of reliable genome-based tools for transcript imaging such as microarrays or qPCR panels. Recently, the genome of the Chinese hamster *Cricetulus griseus* derived cell line (CHO)-K1 was published because it is the preferred host cell line for industrial production of recombinant proteins such as therapeutic antibodies [6]. For this reason, genome analysis and annotation was focused on the analysis of protein modifying systems such as fucosylation or glycosylation pathways. A partial transcriptome analysis of an antibody producing CHO line by RNA sequencing had been performed earlier [7]. The RNA source, the low transcript annotation coverage of approximately 40% combined with the absence of data in the public domain limits utility of this study for our project. Furthermore, a recent mitochondrial genome based study of rodent phylogeny shows that evolutionary relationship between *M*. *auratus* and *C*. *griseus* is comparable to that between *R*. *norvegicus* and *M*.*musculus*[8]. These circumstances suggested use of well annotated genomes such as rat, mouse or human as template for mapping and reliable annotation of Syrian hamster liver transcripts.

We show here, that simultaneous application of two independent deep sequencing technologies combined with searches across multiple database allows indeed unambiguous annotation and quantification of transcripts expressed in liver of the Syrian hamster. Triple match sequencing (TMS) can be applied for transcript profiling of any organism provided that annotated genomes of related species are available.

## Results

### Experimental strategy

**Figure 1:**
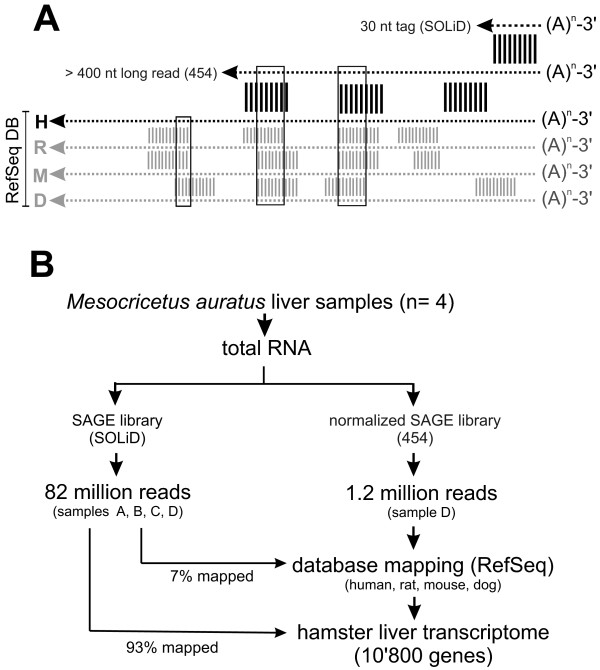
**Triple match sequencing** (**TMS**): **principle and workflow.** (**A**) A complex SAGE-tag based deep sequencing library is generated from total RNA and sequenced on a high-throughput platform (e.g. ABI-SOLiD, ABI-Proton, Illumina) for transcript quantification based on read frequency. A second normalized SAGE library from the same RNA and sequenced on a long read, low-throughput platform (e.g. Roche 454). mRNA reads from these libraries share the distal 3^′^-sequence. The long reads are compared to related transcript databases (i.e. human, mouse, rat, dog; RefSeq) as detailed in Methods. (**B**) Deep sequencing libraries from total RNA were constructed for either SOLiD or 454 sequencing platforms. To ensure representation of low-abundance transcripts it is essential to normalize the 454 library by competitive hybridization. The long 454 reads are assembled into contigs and mapped to human, rat, mouse and dog RefSeq databases yielding about 10’800 annotated genes (hamster liver transcriptome). 93% of 82 million short SAGE tags mapped to the hamster transcriptome and the remaining 7% to human RefSeq entries allowing quantification over 4 orders of magnitude. All 454 and SOLiD sequencing data generated here are available on request.

The underlying principle and the workflow of TMS are summarized in Figure 1 (A and B). From total RNA, two libraries are constructed for NGS using a long-read (Roche 454) and a short-read (ABI SOLiD) technology. For the 454 library construction, we used polyT-primed cDNA because the 3^′^ sequence upstream of the poly-A tail is required for quantification of short-read tags. This approach allows identification of genes belonging to homologous families because the untranslated region has in general higher sequence diversity than the coding region. To achieve broad representation of transcripts independent of their expression levels it was crucial to normalize RNA abundance in the cDNA samples by competitive hybridization. 1.2 million 454 reads with an average length of 350 bases were assembled into contigs. Unique contigs together with unassembled reads were subsequently used as query for homology searches across well annotated transcriptome databases such as mouse, rat and human (RefSeq). This approach led to unambiguous annotation of 10’800 transcripts corresponding to 57.4 per cent of the contigs assembled from the normalized 454 long-read library. Probing of human, mouse and rat RefSeq databases with unique query contigs typically produced matches to the same transcript in each library thereby improving annotation fidelity of hamster transcripts.

Our primary objective is to quantify hamster liver mRNA abundance for interspecies comparisons (see below). Using total liver RNA from the same samples sequenced by 454 technology, we generated SAGE short read libraries for processing on an ABI-SOLiD instrument. We relied on SAGE based bead libraries because the frequency of unique tags is directly proportional to transcript abundance [9]. We performed four SOLiD runs yielding altogether 82 million usable reads. 93% of this pool mapped to the 454-contig assembly and the remainder mapped either to human, mouse, rat, or dog RefSeq databases (Figure 1B). The high mapping efficiency supports the TMS approach and highlights the requirement of a long-read library for reliable mapping and annotation.

### Quantitative comparison of transcripts involved in lipid metabolism in liver of H.sapiens, M.fascicularis, S.scrofa, M.auratus and R.norwegicus

**Figure 2:**
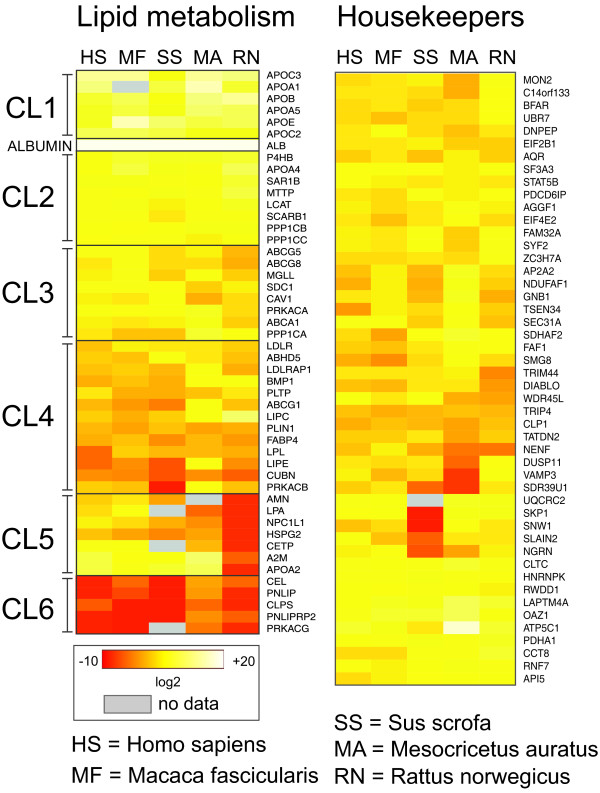
**RNAseq** (**SAGE**) **based comparative analysis of gene expression in liver tissue of H**. **sapiens**, **M**. **fascicularis**, **S**. **scrofa**, **M**. **auratus and R**. **norwegicus.** 48 genes from the public domain network REACTOME “Lipid digestion, mobilization, and transport” were selected to highlight species specific differences in gene expression relevant for HDL biosynthesis (left panel). Gene clusters generated by hierarchical clustering are labelled as CL1 to CL6. The highly abundant albumin transcript is marked. The right panel shows mRNA levels of a standard reference set of liver housekeeping genes compiled from public domain data (L. Badi, unpublished). The log2 values of normalized read counts are presented in a standard heat map as indicated. Grey fields indicate genes lacking valid expression data. SAGE tags of cluster 6 transcripts were quantified using the Chinese hamster genome draft as resource because they had no matches in the 454 library due to low abundance. The RNA source for each species is given in the material section of the paper. All SAGE libraries were built using commercial kits and the source of tissue is given under Methods. Abbreviations of organisms included are indicated at the bottom.

The primary motivation for our study was to compare liver gene gene expression in hamster with humans and model organisms used in metabolic disease research. For this study we constructed SAGE-based liver RNA libraries from *H*. *sapiens*, the minipig *S*. *scrufa* and the rat *R*. *norwegicus*. Human and rat sequence-tags were annotated and quantified by mapping to public domain genomes or to a minipig draft genome assembly [R. Schmucki, unpublished data]. *M*. *fascicularis* liver expression data came from a previous study [5]. The general concordance of the normalized datasets for each species is illustrated by the comparable expression levels of housekeeping genes across all species included in this deep sequencing based analysis (Figure 2, right panel). To compare expression of genes involved in lipid digestion, mobilization and transport we selected a set of 48 genes from the REACTOME database (http://www.reactome.org/), because their functional interactions are well documented. Hierarchical clustering generated six distinct clusters of genes with similar gene expression patterns across all species (Figure 2; left panel; CL1- CL6). Cluster 1 contains apolipoprotein isoforms that are abundantly expressed in all species included here. ApoA2 was assigned to cluster five which contains transcripts involved in HDL-biosynthesis with very low expression in the rat. The cholesteryl-ester- transfer-protein (CETP) is the target of HDL-modifying drugs explaining lack of pharmacological responses in the rat model [10]. Cluster 2 contains highly expressed transcripts in all species adjacent to the most abundant liver specific transcript albumin. Clusters 3 and 4 are compiled of genes in the intermediate expression range, with notable interspecies variability. Finally, the mRNA expression levels in cluster 6 are in general low because these genes mostly belong to a network operating in the intestinal lumen. The protein kinase PRKACG as a testis-specific enzyme was properly assigned to cluster 6 [11]. Altogether we failed to identify transcripts for five genes (Figure 2, left panel, grey boxes). The ApoA1 gene of *M*. *fascicularis* lacks a CATG motif required for SAGE-based sequencing using SOLiD technology. Three *S*. *scrofa* genes (PRKACG, CETP, LPA) are missing in the draft genomes of the pig [12] or the minipig (R. Schmucki, unpublished data). The *M*.*auratus* AMN receptor gene (cluster 5) was not present in our long read library.

## Discussion

We show that triple match sequencing (TMS) allows robust deep sequencing based transcript imaging in absence of an annotated genome. Using a SAGE based comparison of liver gene expression across five species we show that TMS derived data are comparable to mRNA quantification data using conventional genome based mapping. Similar to other vertebrates about 10’800 genes are expressed in hamster liver at comparable levels (Additional file 1: Table S1).

The value of transcript identification in absence of an annotated genome has been recognized by others. Fletcher et al. have generated a partial transcriptome of the Woodchuck (*Marmota monax*) an animal model for experimental hepatitis B infection and hepatocellular carcinoma [7]. Randomly primed cDNA libraries were sequenced using 454-long read technology followed by contig assembly and annotation. This yielded a pool of 13’448 unique transcripts which was used to build a custom microarray to analyze the transcriptional responses to virus infection and cure. Compared to TMS this process is more time consuming due to the need of microarray production and validation. A method termed TRINITY relies on graphics based assembly of high coverage short read or paired end cDNA libraries without reference genome [13]. In contrast to the Woodchuck approach, the three software modules constituting TRINITY can detect novel splice variants as demonstrated for fission yeast, mouse, and whitefly whose genomes were not available at the time of release. This approach is particularly suited for the analysis of tumor samples where gene re-arrangements and structural deviations can lead to novel variants of any gene [14]. Although TRINITY has powerful transcript assembly capabilities for short and long read data the current version of the program cannot perform transcript quantification. In our approach contig assembly of long-reads is solely performed for annotation followed by quantification using frequencies of short-read SAGE tags (Figure 1A). Therefore, TMS is inherently unable to quantify or discover splice variants of the same gene. This requires construction of tissue specific cDNA libraries by random priming which was used to build the human “bodymap” gene expression database (http://www.ensembl.info/blog/2011/05/24/human-bodymap-2-0-data-from-illumina). TMS does not attempt to annotate species-specific transcripts because the approach relies on mapping to known mRNAs in other species. We note that any unmapped long reads are candidates for species specific transcripts of hamster. Normally, unique 454- reads or contigs matched the same RefSeq transcript from more than one species (Figure 1B) resulting in a significant improvement of annotation confidence.

As it turned out, the genome sequence or the partial transcriptome analysis of the *C*. *griseus* derived cell line CHO(K1) had only limited value for our project [6,15], because the annotation efforts in *C*. *griseus* focused mainly on pathways relevant for protein production and modification. Furthermore, the evolutionary distance between *C*. *griseus* and *M*.*auratus* is comparable to that between mouse and rat [8]. Finally, the partial transcriptome of CHO is derived from an ovary cell line which expresses probably different genes than liver tissue from another species.

## Conclusions

Triple match sequencing is a robust and rapid method for annotation and quantification of transcripts in the absence of a reference genome. The method is especially useful in projects where reliable mRNA quantification rather than primary sequence information is important. TMS does not require implementation of specialized hard- or software which makes it fast, affordable and user friendly. It might even contribute to the identification and selection of novel animal models prior to a genome sequencing effort.

## Methods

### Animals

Male Syrian hamsters, weighing 125 ± 5 g were obtained from Charles River Germany (Sulzfeld). The animals were kept at standard housing conditions (22±2°C, 50-60% humidity with a range of 40-80% and 12/12 light/dark cycle). They were supplied with standard laboratory chow and water ad libitum, and left to acclimatize for 1 week before the experiments. The harvested liver was cut in small pieces (approximately 3 × 3 mm) and snap frozen immediately for further processing. All experimental procedures were carried out in accordance with international guidelines for care and use of laboratory animals.

### RNA isolation

RNA was isolated from Syrian hamster liver samples using RNeasy Mini Kit from Qiagen (No.74104). RNA integrity was analyzed using an Agilent Bioanalyzer (RNA 6000 Nano Kit) and all samples reached a quality score (RIN) above 9.7. Total Wistar rat liver RNA was a gift from Dr T. Heckel and snap-frozen minipig liver tissue was a gift from Dr Niels-Christian Ganderup (http://www.ellegaard.com). Normal human liver RNA was provided by the Human Tissue and Cell Research foundation (project-nr: 2012–13 approved 06/25/2012).

### Construction of SAGE based libraries and NGS procedures

The Applied Biosystems SOLID 3 System SAGE kit (No.4443756AB) was used for library construction. Presence of the expected 100-base pair SAGE templates was confirmed using an Agilent Bioanalyzer (High Sensitivity DNA Assay). The emulsion PCR (ePCR) library was constructed using DNA at a concentration 0.75 pM. The ePCR was carried out on a SOLID EZ Bead amplifier and the enriched beads were quantitated using a NanoDrop 2000 spectrophotometer. Sequencing was performed on a SOLiD4 instrument with DOLF-TOP sequencing chemistry Frag-Lib F3 Tag MM35 (No. 445352).

### cDNA normalisation for GS FLX (Roche 454) libraries

To normalize the levels of cDNA prior to library construction we used a competitive hybridization protocol provided with commercial kits (Evrogen MINT cDNA synthesis kit No. SK001; TRIMMER cDNA Normalization kit (No. NK001). For 454 sequencing the use of special cDNA primers is required. The 3^′^-kit primer is replaced by polTdeg (5^′^-AAG CAG TGG TAT CAA CGC AGA GTA CTT TTG TTT TTT TTT CTT TTT TTT TTV N -3^′^) The kit PCR primer M1 is substituted by polTM1 (5^′^-AAG CAG TGG TAT CAA CGC AGA GTA CGG-3^′^). The product of the full-size preparation of ds cDNA is used for normalization with the TRIMMER kit using 700-1300 ng of purified cDNA. The normalization is performed according to the supplied protocol without any changes. emPCR was carried out with 5cpb input on SOLID EZ Bead Amplifier. Typically a GS FLX+ Sequencing Run generated about 800’000 reads with an average length of 410 bases.

### Raw sequencing data processing, read mapping, contig assembly and gene annotation

Following depletion of ribosomal sequences, 454-sequencing reads were assembled *de novo* using the software Trinity with the option “inchworm” as assembly method (release 2012-01-25) [13]. Unassembled reads of low complexity or shorter than 100 bp were removed, and the remainder was merged with the assemblies to produce a set of *bona fide* transcripts. Re-aligning the raw reads to the set of transcripts with Roche-454’s Newbler software (version 2.7) yielded a mapping efficiency of 97%.

To annotate contigs with gene symbols, BLAST [16] mapping to reference transcript databases (RefSeq human, mouse, and rat) were performed. Only contig matches with E-values lower than 1.e-4 were considered for annotation. This cutoff is less stringent and an adaptation for mapping of the less conserved 3^′^-UTRs (see text and Figure 1A). Sequence identities of these matches were determined by pair-wise Smith-Waterman alignment of the contig sequence with the corresponding transcript sequence. An Elbow criterion was applied to determine the alignment with highest sequence identity and to annotate the contig sequence accordingly.

Reads derived from the SAGE library were mapped to the transcripts described above using ABI’s SOLiD SAGE software (version 1.10) with the tag length set to 22 bases and a maximum of two mismatches allowed. The read frequencies per transcript for all species included in this study are available in Additional file 1: Table S1.

## Abbreviations

SAGE: Serial analysis of gene expression; CDS: Protein coding sequence; NGS: Next generation sequencing; emPCR: Emulsion polymerase chain reaction; qPCR: Quantitative polymerase chain reaction

## Competing interests

The authors declare that they have no competing interests.

## Authors’ contributions

RS and MB carried out deep sequencing data processing and transcript analysis and quantification. EK performed library construction and sample normalization. SG provided liver samples from matched hamsters. SL and WT selected a well annotated human liver sample and provided high-quality total RNA. ME designed the workflow and experimental strategy. UC lead the project and drafted the manuscript. All authors have read and approved the final version of this manuscript.

## Supplementary Material

Additional file 1: Table 1 Read frequencies of transcripts detected in human, cynomolgus, minipig hamster and rat liver samples. Given are the absolute read frequencies without normalization for the species indicated using standard gene symbols. Click here for file
